# Improving the Anti-Corrosion and Anti-Wear Performance of Anodic Coating on the Surface of AA 5052 via Hydro-Thermal Treatment

**DOI:** 10.3390/ma15041447

**Published:** 2022-02-15

**Authors:** Debo Liu, Baofeng Zhang, Wei Song

**Affiliations:** 1 Faculty of Engineering, Huanghe Science and Technology College, NO.666 Zijingshan South Road, Zhengzhou 450063, China; hkdgxbjj@163.com (D.L.); zgwang2005@sohu.com (B.Z.); 2 School of Biological and Chemical Engineering, Nanyang Institute of Technology, NO.80 Changjiang Road, Nanyang 473004, China; 3 Faculty of Materials Science and Engineering, Xi’an University of Technology, NO.5 South Jinhua Road, Xi’an 710048, China

**Keywords:** anodizing, hydro-thermal technology, anti-corrosion, anti-wear, aluminium alloy

## Abstract

Hydro-thermal technology had been used to improve the anti-corrosion and anti-wear performance of anodizing coating on the surface of aluminium alloys. The micromorphology of the coating has been studied by SEM and results proved the coating had a compact structure. The element in the substrate had been characterized by EDS and results proved Fe had redissolved to the Al substrate. The crystalline structure of the coating had been studied by XRD and results proved the anodic coating could be transformed into η-, p- and γ-alumina. The electrochemical properties had been researched using an electrochemical workstation; results proved after the coating had been treated by hydro-thermal technology, its anti-corrosion properties could be improved. At the hydro-thermal temperature of 400 ℃, its open circuit voltage and impedance reached −0.46 V and 160 kΩ × cm^2^, respectively. The hardness of the coating had to be measured with an HVS-100 micro-hardness tester, with results proving that, after the hydro-thermal treatment, the hardness of the coating increased to 150 HV. The friction coefficient of the coating had been studied using a ball-on-disk tester, and the results proved it decreased to 0.46. The MMW-2 scratch tester had been used to measure the adhesion between the coating and substrate; results proved the coating had better adhesion with the substrate. The thermal conductivity of the coating had been studied by a heat conduction coefficient measurement device; results proved that it reached 11.2 W/m × K at a hydro-thermal temperature of 400 ℃, far higher than that of organic coating.

## 1. Introduction

Aluminium alloys have high heat conductivity, high specific strength, and no low-temperature brittleness. It has become the preferred material in cryogenic heat exchangers [1,2,3,4], but the poor corrosion resistance and wear resistance shorten its service life, especially when the hot/cool fluid is an acid or alkaline solution. Although painting various organic coatings on its surface can improve its corrosion resistance, the low thermal conductivity of the coating decreases the heat exchange efficiency. Using an anodizing method [5,6,7], a uniform alumina oxide can be prepared on the surface of the aluminium alloy. However, the coating is porous, its corrosion resistance is poor, and can only protect the substrate in the short-term [8,9,10]. To improve the anti-corrosion of the coating, usually, the through pore needs to be sealed. Because the volume of hydrated alumina is larger than that of alumina oxide, it has become a conventional treatment to immerse the anodic coating in hot water at 90–95 °C. However, the coating consists of amorphous alumina, meaning it can be easily eroded [11,12,13]. Besides, its thermal conductivity [14] (0.2–3 W/m × K) is far lower than that of the aluminium alloy (236 W/m × K), which limits its application as a heat exchanger.

It is well known that crystalline alumina oxide has higher thermal conductivity, anti-corrosion, and anti-wear performances than that of amorphous alumina [15,16,17]. If the amorphous alumina can be transformed into crystalline alumina, its anti-corrosion and anti-wear properties can be improved. According to the transition relationship of alumina and its hydrate, the amorphous alumina oxide can be transformed into crystalline alumina. When the temperature reaches 230 °C, 300 °C, and 450 °C, amorphous alumina can be transformed into η-, p- or γ-alumina oxide, respectively [18]. However, with the dehydration of amorphous alumina, an internal stress is produced [19]. Thus, the quantity and size of cracks in the anodic coating increase with the increase in temperature. When the amorphous alumina particle is treated by hydro-thermal technology, it can be transformed into crystalline alumina [20,21,22]. The relationship between hydro-thermal temperature and the crystallinity of alumina particles has been studied by many researchers [23,24,25]. To increase the crystallinity of alumina, the temperature is high [26,27]. Although, to a certain degree, the cracks can be inhibited by hydro-thermal technology. The cracks in the anodic coating are significantly affected by temperature. Its quantity and size increases with the increase in temperature [28,29]. If the anodic coating was treated by hydro-thermal technology at a suitable temperature, a compact crystalline alumina coating could be prepared.

Based on the above analysis, using hydro-thermal technology can improve the anti-corrosion or anti-wear of the anodic coating. However, improving them simultaneously is a challenge. To conquer the problem, the influence of hydro-thermal temperature on the performances of anodic coatings has been studied. The anti-corrosion and anti-wear of AA 5052 was improved. The thermal conductivity of the coating was researched and compared with that of the anodic coating, proving the heat transfer performance of the coating could be improved.

## 2. Experiment

### 2.1. Materials and Pre-Treatment

The 25 × 25 × 2 mm^3^ Al-alloy 5052 (AA 5052) specimens were used to be the substrates in anodizing. Table 1 shows alloy composition.

After the commercial specimens were mechanically ground with 400 and 800 grit silicon carbide, they were washed with distilled water, then washed with deionized water and dried in a cold air stream.

### 2.2. Experimental Process

Each of our experimental reagents was provided by Tianjin Kemiou Chemical Reagent Co. Ltd, Tianjin, China. Two sheets of AISI 321 stainless steel with dimensions of 300 × 300 mm^2^ (Ben Lai Metal, Shanghai, China) were adopted to be the cathode. Using the samples as the anode, the anodizing process was conducted in 150 g/L sulphuric acid solution for 40 min at the current density of 30 mA/cm^2^ and temperature of 4 °C. Thereafter, the samples were treated by hydro-thermal technology at 200, 300, 400 and 450 °C for 2 h.

### 2.3. Characteristic

X-ray diffractometer (XRD, (D/max-rB, RICOH, Tokyo, Japan) with CuKa source was employed to examine the phase compositions for diverse coatings. The current applied and accelerating voltage 30 mA and 40 kV, separately. Scanning electron microscopy (SEM, S-4700, Hitachi, Honshu Island, Japan) was adopted to investigate the cross-section and surface microstructures of the coating. Besides, the energy dispersive spectrometer had been used to characterize the elements on the surface of the substrate. The porosity of the coatings was statistically analyzed by Image J software. The adhesion of the coating was tested by an MMW-2 scratch tester (Shanghai Qianshi precision Electromechanical Technology Co., Ltd., Shanghai, China), acoustic emission test method was used to evaluate the adhesion of the coating. The test condition is the scratch speed of 1 mm/min, scratch length of 10 mm and load rate of 10 N/min. The ball-on-disk tester (Bruker, UMT-Tribolab, Bilerica, MA, USA) was employed to evaluate coating friction coefficient in the case of dry sliding. In this experiment, the GCr15 balls (diameter, 10 mm; hardness, HRC 60) were adopted to be counterface materials. Besides, the linear velocity of sliding was set at 0.01 m/s, with a normal load of 5 N. Each experiment was performed for 30 min in laboratory conditions (temperature, 25 °C; relative humidity RH, 50%). Moreover, in every test, a computer was used to record friction coefficients. Because the coating was porous, and the hardness of AA 5052 is low, the HVS-100 micro-hardness tester (TMVS-1, TIMES Group, Beijing, China) with a load of 100 g for 10 s was used to evaluate the micro-hardness of the coating at 25 °C and relative humidity RH of 50%. The open circuit voltage and electrochemical impedance of the coating was measured and analyzed by the electrochemical workstation (PARSTAT 4000, Princeton Applied Research, Princeton, NJ, USA). Furthermore, three electrode systems were used, the working electrode was the coatings. The counter electrode was a platinum sheet, while saturated calomel served as the reference electrode. The contact area between the sample and the solution was 1 cm^2^, and 3.5 wt.% sodium chloride was used as a solution. The starting and ending frequencies were 100 kHz and 0.01 Hz, respectively. After testing, relevant information on the coating was obtained by fitting the ZsimpWin V 3.6 software. Thermal conductivity was measured by pulse method, with a heat conduction coefficient measurement device (TC 3000 E, XIATECH Electronic Technology Co., Ltd., Xi’an, China) at 25 °C for 0.9–1.2 s.

## 3. Results and Discussion

### 3.1. Micromorphology

**Figure 1:**
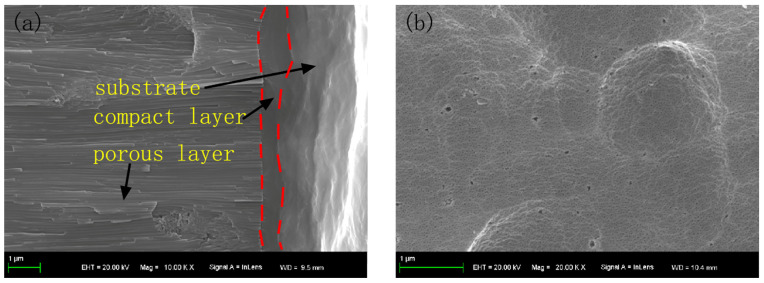
The (**a**) cross-section and (**b**) micro surface of the anodic coating.

At high magnification, the cross-section and micro surface of the anodic coating were observed and are illustrated in Figure 1. It can be seen from Figure 1a that, on the surface of the aluminium alloy, there was a compact layer and a porous layer [30]. It can be seen from Figure 1b that the pores are evenly distributed on the surface of the aluminum alloy. However, due to capillary action, the solution concentration in the pore was higher than outside the pore. When the sample was placed in a corrosive solution, the anodic coating was easily corroded.

**Figure 2:**
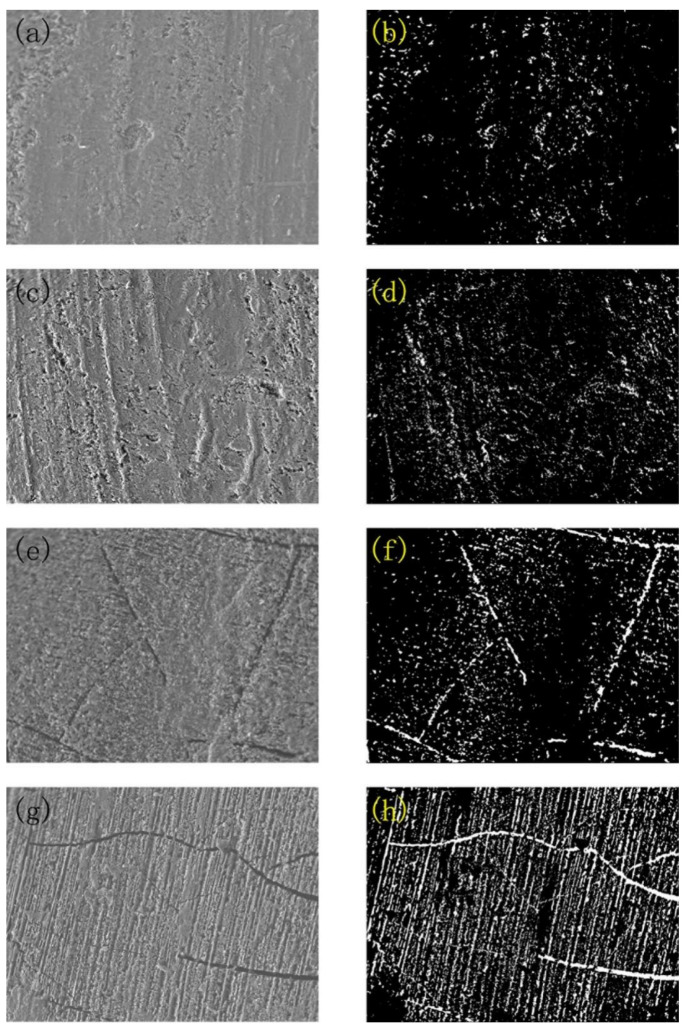
Micro surface structure of SEM image and its porosity analysis by Image J software on anodic coating treated by hydro-thermal technology at (**a**,**b**) 200 °C, (**c**,**d**) 300 °C, (**e**,**f**) 400 °C, and (**g**,**h**) 500 °C.

Figure 2 illustrates the micro surface structure and its porosity photograph of the anodic coating treated at different temperatures. It can be seen that with the increase in hydro-thermal temperature, the quantity and size of cracks on the surface increases. Besides, the porosity of the coatings increases with the increase in hydro-thermal temperature. The main reason is that of temperature; the amorphous alumina was transformed into a crystalline structure, and the bound water contained in the amorphous alumina was released. The water in the pore was gasified at high temperatures and the volume increased [31]. After the hydro-thermal treatment was completed, the water vapor in the pore was liquefied and the volume decreases. Because the volume of crystalline alumina is smaller than that of hydrated alumina [32], micro cracks were produced in the coating due to dehydration force.

### 3.2. EDS Analysis

**Figure 3:**
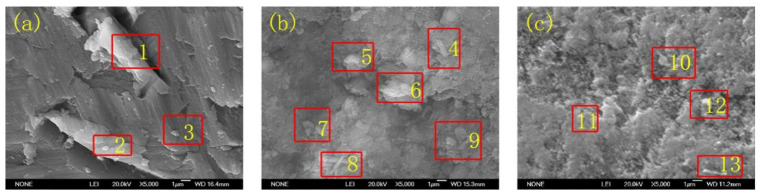
SEM image of (**a**) AA 5052, etching morphology of (**b**) bare surface and (**c**) after being treated by hydro-thermal technology at 400 °C for 2 h (the coating was stripped). Notes: the etchings were performed in 20 wt.% HNO_3_ solution. Before the etching, the treated coatings were stripped from the substrate.

Figure 3 illustrates the morphology of bare AA 5052 and its etching image. Besides, the micro morphology of the substrates, after being treated by hydro-thermal technology, was observed. The element composition at different positions is listed in Table 2. It can be seen that either Al_3_Mg_2_, AlCu_2_, or Al-4Mg-Fe-Mn was in the substrate. After the substrate had been etched, Al-Cu_x_-Mn_y_-O_z_, Al-Fe_x_-Mn_y_-O_z_, Al-Mg_x_-Fe_y_-O_z_, Al-Cu_x_-Mn_y_-O_z_, Al_x_-Mg_y_-O_z_ were observed. The anti-corrosion of the aluminium alloy is significantly affected by the alloying element. The pitting of AA 5052 occurred near the second phase products. Meanwhile, after the substrate had been treated by hydro-thermal technology, the Fe was redissolved into the substrate. Because the pitting of AA 5052 is mainly caused by Fe, its pitting had been improved. It can also be seen from Figure 3b,c that the grain size became smaller, which was also helpful in improving the anti-corrosion properties.

### 3.3. Crystalline Structure

**Figure 4:**
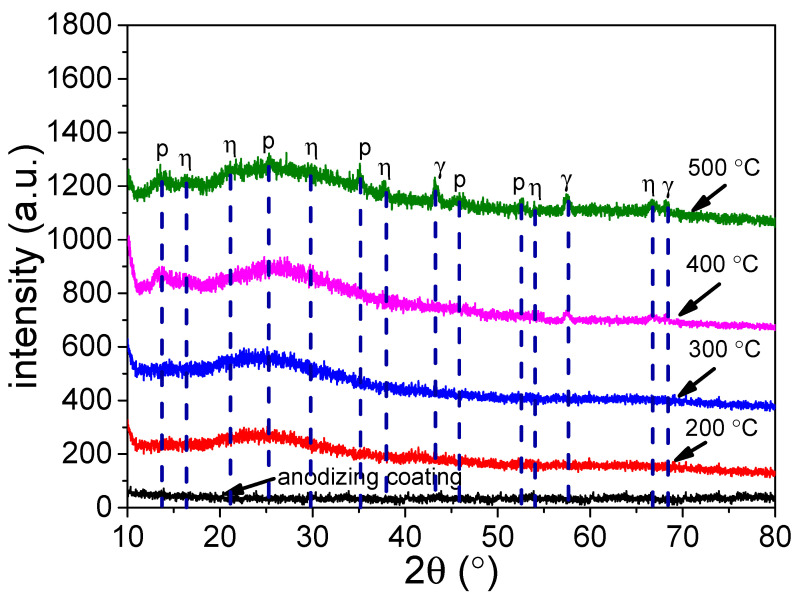
XRD spectra of anodic coating before and after being treated by hydro-thermal technology at 200 °C, 300 °C, 400 °C and 500 °C.

The crystalline structure of the samples was analyzed by X-ray diffractometer. Figure 4 illustrates the influence of hydro-thermal temperature on the crystalline structure of the coating. It can be seen that the anodic coating is an amorphous structure. After being treated by the hydro-thermal technology, the diffraction peaks of alumina appeared, proving the amorphous alumina can be transformed into crystalline alumina. Besides, the peak intensity of the coating increased with the increase in temperature. It can also be seen that when the temperature was 200 °C, the amorphous alumina could not be transformed into crystalline alumina. When the temperature reached 300 °C, the amorphous alumina could be transformed into η- and p- crystalline alumina [33]. When the temperature reached 400 °C, the characteristic peak of γ-alumina could be observed [34]. The results are in agreement with the crystalline phase transition temperature of hydrated alumina [35].

### 3.4. Electrochemical Corrosion Behavior

**Figure 5:**
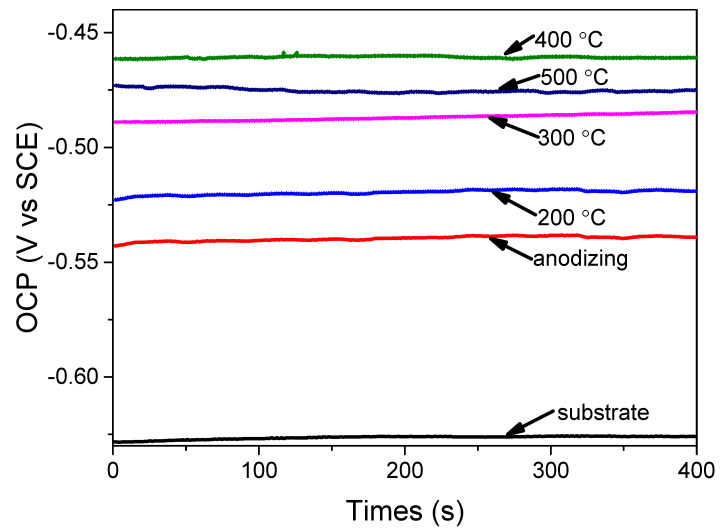
The open circuit voltage of Al substrate, anodic coating before and after being treated at 200 °C, 300 °C, 400 °C, and 500 °C.

**Figure 6:**
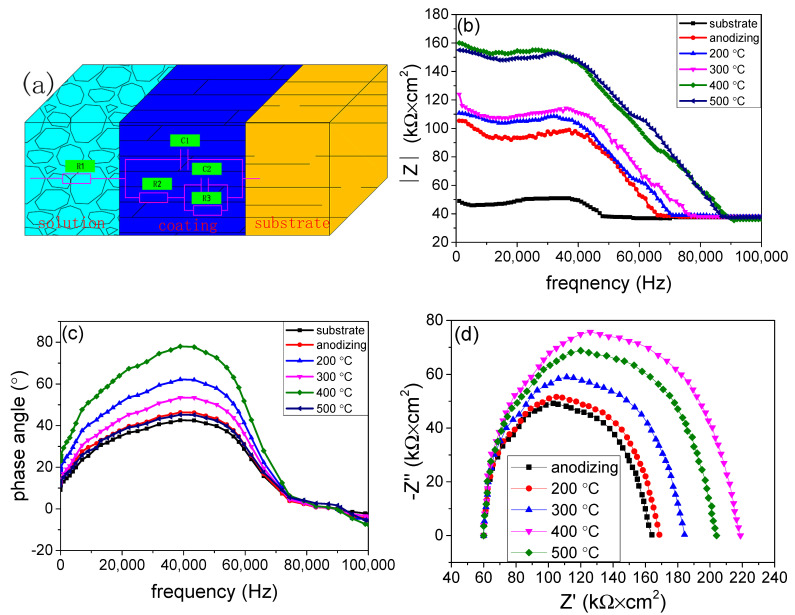
The (**a**) equivalent circuit diagram, (**b**) Bode impedance–frequency plot, (**c**) Bode phase angle–frequency plot and (**d**) Nyquist plot of Al substrate, anodic coating before and after being treated at 200 °C, 300 °C, 400 °C, and 500 °C. Note: before being characterized, all the samples were immersed in 3.5 wt.% NaCl solution for 1 day at room temperature.

After the samples were immersed in 3.5 wt.% NaCl for 1 day, their open circuit voltage was measured and is illustrated in Figure 5. It can be seen that the open circuit voltage of the AA 5052 substrate was about −0.66 V, after it had been treated by anodizing and hydro-thermal technology, and the open circuit voltage increased. It is a fact that low open circuit potential means a high tendency to corrode. When the anodic coating had been treated by hydro-thermal technology at 400 °C, its open circuit voltage was about −4.6 V, proving it had better corrosion resistance. At the same time, the AC impedance test was performed. The equivalent circuit diagram of the coatings was proposed and is shown in Figure 6a. In the equivalent circuit, R1 represented solution resistant, R2 represented porous coating capacitive reactance, R3 represented compact coating capacitive reactance, while C1 represented porous coating inductive reactance, and C2 represented compact coating inductive reactance. The Bode impedance–frequency plot, Bode phase angle–frequency plot and Nyquist plot are shown in Figure 6b, Figure 6c and Figure 6d, respectively. It can be seen from Figure 6b that after the substrate had been treated, its total impedance modulus increased significantly, proving the anti-corrosion of AA 5052 had been improved. This is because the initial impedance modulus in the high frequency (<40 kHz) region is related to the coating properties. When the anodic coating had been treated at 400 °C, its impedance reached 160 Kω × cm^2^, showing a good protection for the substrate. This is due to the curve at medium frequency (1–10^3^ Hz) regions in the Bode phase angle–frequency plot, related to the coating properties. Besides, the anti-corrosion of the coating increased with the increase in phase angle. It can be seen from Figure 6c that after the substrate had been treated, its phase angle increased, proving the anti-corrosion had been improved. It can be seen from Figure 6d that the impedance of the anodic coating was about 105 kΩ × cm^2^. After the coating had been treated by hydro-thermal technology at 400 °C, its impedance reached 160 kΩ × cm^2^. Evidently, the anodic coating, after being treated at 400 °C, presented a maximum radius among all the samples, suggesting the highest anti-corrosion properties. The main reason is that the impedance of the anodic coating is affected by its crystallinity and compactness. The crystallinity increased with the increase in temperature. However, the compactness decreased with the increase in temperature. When the temperature was 400 °C, there were few cracks in the coating, while its crystallinity was high. Therefore, the coating’s impedance was the highest.

### 3.5. Hardness

**Figure 7:**
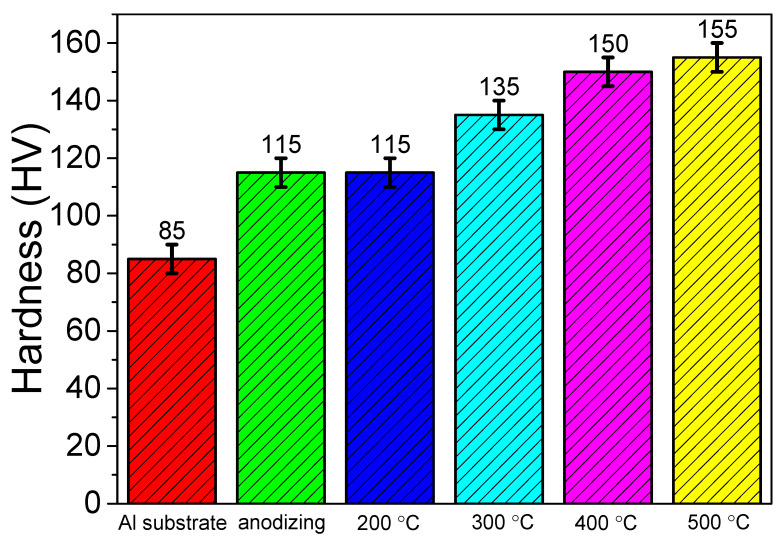
The hardness of anodic coating before and after being treated by hydro-thermal technology at 200 °C, 300 °C, 400 °C and 500 °C.

It can be seen from Figure 7 that the hardness of AA 5052 was only 85 HV [36], while the hardness of the anodic coating was 115 HV. After the anodic coating was treated by hydro-thermal technology, the hardness of the coating increased. When the temperature increased to 500 °C, the hardness of the coating reached 155 HV. The main reason was that the hardness of the coating increased with its crystallinity, which is affected by the hydro-thermal temperature. This is because the proportion of crystalline alumina in the coating increased with the increase in hydro-thermal temperature. Therefore, the hardness of the coating prepared at 500 °C was the highest.

### 3.6. Friction Coefficient

**Figure 8:**
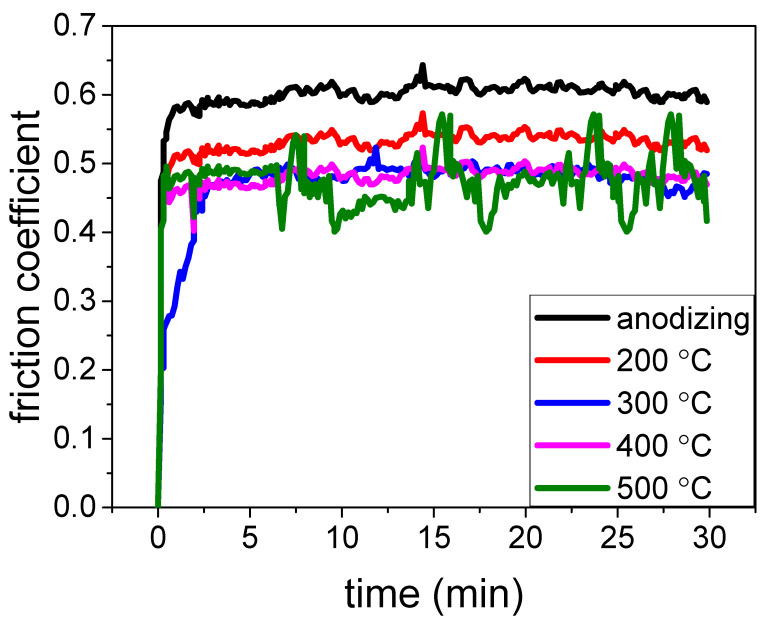
Friction coefficient of Al-alloy, anodic coating before and after being treated by hydro-thermal technology at 200 °C, 300 °C, 400 °C and 500 °C.

For the heat exchange pipe suffering the erosion of hot and cool fluid, decreasing the friction coefficient is helpful to decrease its erosion and extend its service life. Thus, the friction coefficient of samples has been studied. Figure 8 illustrates the friction coefficient of the anodic coating treated at different hydro-thermal temperatures. It can be seen that the friction coefficient of the anodic coating was about 0.62 [37]. After the coating had been treated by hydro-thermal technology, the friction coefficient decreased. When the hydro-thermal temperature reached 400 °C, the friction coefficient of the coating decreased to about 0.46. When the temperature was 500 °C, the friction coefficient maintained at about 0.46. However, the variation in the friction coefficient at different times increased. The results show that the friction coefficient of the coating can be improved by hydro-thermal technology, and the suitable hydro-thermal temperature was 400 °C.

### 3.7. Adhesion

**Figure 9:**
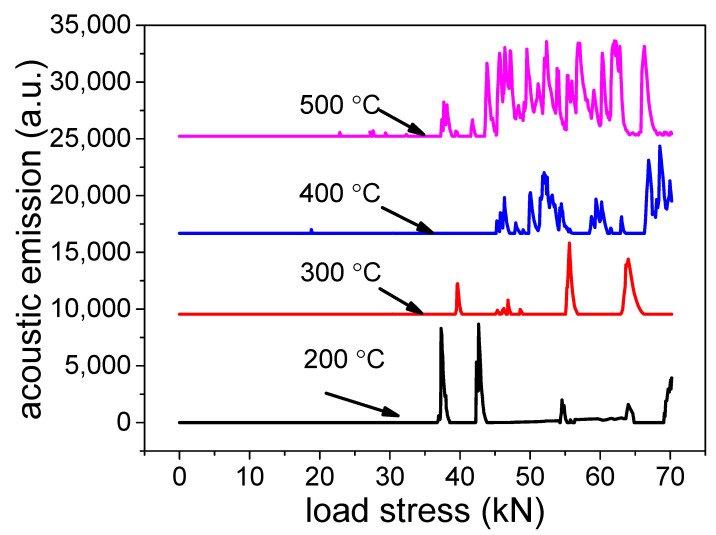
Adhesion between coatings and substrate.

Good adhesion contributes to improving the thermal conductivity and preventing the coating separating from the aluminum alloy substrate [38]. Figure 9 illustrates the adhesion between coatings and substrates. It can be seen that when load stress reached 38 N, the treated coating at 200 °C was crushed. Besides, the load stress increased with the temperature increase. When the hydro-thermal temperature was 400 °C, the adhesion between the coating and substrate reached 44.5 kN, except for the occasional small acoustic emission intensity, surveyed at low load stress. The main reason is that both hardness and microcrack affect the load stress. When the hydro-thermal temperature was 200 °C, the coating was amorphous. When the temperature was 500 °C, many microcracks appear in the coating. Both amorphous alumina with low hardness and too much crack in the coating tended to be destroyed during the scratch test.

### 3.8. Thermal Conductivity

**Figure 10:**
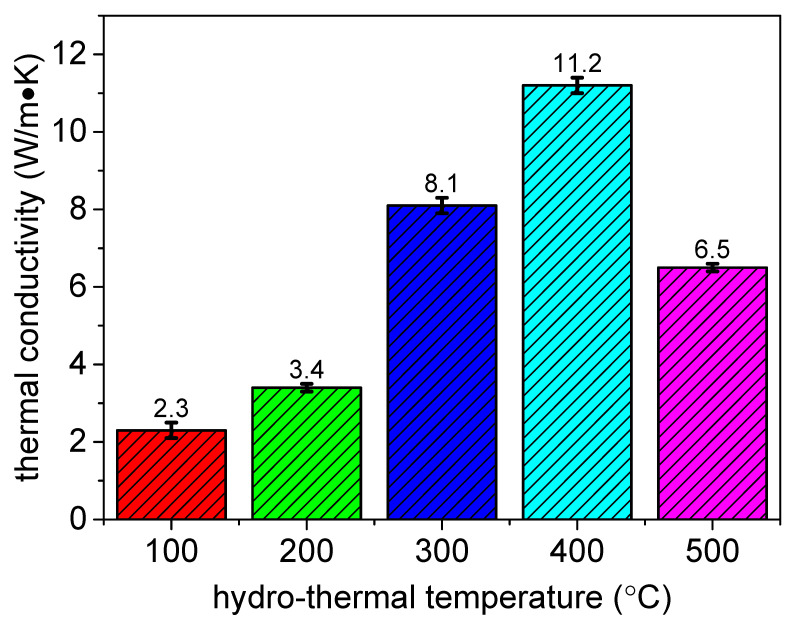
Thermal conductivity of anodic coating before and after being treated by hydro-thermal technology at 200 °C, 300 °C, 400 °C, and 500 °C.

The thermal conductivity of the samples is indicated in Figure 10. It can be seen that the thermal conductivity of the anodic coating was 2.3 W/m × K. After being treated by hydro-thermal technology, the thermal conductivity of the coating increased. Besides, the coating had the highest thermal conductivity when the hydro-thermal temperature was 400 °C. This is because the thermal conductivity of the coating is affected by its crystalline structure and compactness. When the hydro-thermal temperature was 400 °C, a compact crystalline alumina coating could be prepared. Thus, the heat transfer performance of the anodic coating can be improved.

For the cryogenic heat exchanger, suffering from corrosion and erosion, improving its anti-corrosion and anti-wear properties is key to prolonging its service life. Using hydro-thermal technology, the pores in the anodic coating can be sealed at a low temperature (90–95 °C). The amorphous alumina particle can also be transformed into crystalline alumina at a high temperature (>600 °C). To improve both of them simultaneously, at medium temperature, the anodic coating was treated by hydro-thermal technology. The amorphous alumina had been transformed into η-, p- or γ-alumina, and the cracks in the coating were few. Compared with the anodic coating, its open circuit voltage had been increased to −0.46 V, while its impedance had been increased to 160 kΩ × cm^2^, proving the anti-corrosion of the coating had been improved. The hardness had been increased to 150 HV, the friction coefficient had been decreased to 0.46, proving the anti-wear had been improved. Besides, the thermal conductivity had been increased to 11.2 W/m × K, and the adhesion between the coating and substrate was 44.5 KN, proving the coating had a better heat transfer performance.

## 4. Conclusions

A compact crystalline alumina coating had been prepared on the surface of AA 5052, by combining anodizing and hydro-thermal technology. The influence of hydro-thermal temperature on the coating performance was studied. After the anodic coating was treated at 400 °C for 2 h, the amorphous alumina in the coating could be transformed into η-, p- or γ-alumina. Its hardness and friction coefficients were 150 HV and 0.46, respectively. The open circuit voltage and impedance of the coating were −0.46 V, and 160 kΩ × cm^2^, respectively. The results proved that the anti-corrosion and anti-wear of the coating had been improved. Besides, the thermal conductivity and adhesion between the coating and substrate was higher than that of the anodic coating. The coating was suitable for protecting the substrate in the cryogenic heat exchanger.

However, limited by the liquid temperature of the aluminium alloy, the crystalline alumina is hydrated alumina. Besides, there were a few cracks in the coating, which limits the increase in hydro-thermal temperature and the prolonging of treatment time. To further improve the anti-corrosion and anti-wear properties of the coating, inhibiting the growth of cracks in the coating became an important problem to be solved. The cracks are produced by internal stress during the dehydration process. It is well known that the dehydration rate is affected by the heating rate. Thus, we studied the relationship between the pore structure, dehydration rate and the heating rate, which is of benefit, to further improve the coating performance.

## Figures and Tables

**Table 1:** The composition of aluminum 5052 alloy.

Composition	Al	Mg	Si	Cu	Cr	Fe	Mn	Zn	Others
wt%	Balance	2.2–2.8	0.25	0.1	0.15–0.35	0.4	0.1	0.1	0.15

**Table 2:** The elements composition at different positions.

Point	Element Content (wt.%)						
	Al	Cu	Fe	Mn	Mg	Si	O
1	69.23		3.41	9.43	12.94		
2	71.32				28.68		
3	70.56	9.79			19.65		
4	68.22	14.74		12.81			4.23
5	77.33		11.05	2.87			8.65
6	63.35		19.35		5.06		12.24
7	80.95		9.02	0.79		3.60	5.64
8	49.46	32.79	6.68	2.93			8.14
9	61.46	28.17					10.37
10	56.64				23.32		20.04
11	73.21	26.79					
12	68.33				18.02		13.65
13	63.13	20.51		1.12			15.24

## Data Availability

The data that support the findings of this study are available on request from the corresponding author.
